# Genome-wide analyses in Lyme borreliosis: identification of a genetic variant associated with disease susceptibility and its immunological implications

**DOI:** 10.1186/s12879-024-09217-z

**Published:** 2024-03-21

**Authors:** Hedwig D. Vrijmoeth, Jeanine Ursinus, Javier Botey-Bataller, Yunus Kuijpers, Xiaojing Chu, Freek R. van de Schoor, Brendon P. Scicluna, Cheng-Jian Xu, Mihai G. Netea, Bart Jan Kullberg, Cees C. van den Wijngaard, Yang Li, Joppe W. Hovius, Leo A. B. Joosten

**Affiliations:** 1 Department of Internal Medicine and Radboudumc Center for Infectious Diseases, Radboud University Medical Center, Nijmegen, 6500 HB the Netherlands; 2 Department of Internal Medicine, Division of Infectious Diseases, Amsterdam UMC, Location AMC, University of Amsterdam, P.O. Box 22660, Amsterdam, 1100 DD the Netherlands; 3 Amsterdam Institute for Infection and Immunity, Amsterdam, the Netherlands; 4 National Institute for Public Health and Environment (RIVM), Center for Infectious Disease Control, Bilthoven, 3720 BA the Netherlands; 5 Department of Computational Biology for Individualised Infection Medicine, Centre for Individualised Infection Medicine, a joint venture between the Hannover Medical School and the Helmholtz Centre for Infection Research, 30625 Hannover, Germany; 6 TWINCORE, Centre for Experimental and Clinical Infection Research, a joint venture between the Hannover Medical School and the Helmholtz Centre for Infection Research, 30625 Hannover, Germany; 7 Department of Applied Biomedical Science, Faculty of Health Sciences, Mater Dei Hospital, University of Malta, MSD 2080 Msida, Malta; 8 Centre for Molecular Medicine and Biobanking, Biomedical Sciences, University of Malta, MSD 2080 Msida, Malta; 9 Department of Immunology and Metabolism, Life and Medical Sciences Institute, University of Bonn, 53113 Bonn, Germany

**Keywords:** Lyme borreliosis, Disease susceptibility, GWAS, Cytokines, mTOR pathway

## Abstract

**Background:**

Genetic variation underly inter-individual variation in host immune responses to infectious diseases, and may affect susceptibility or the course of signs and symptoms.

**Methods:**

We performed genome-wide association studies in a prospective cohort of 1138 patients with physician-confirmed Lyme borreliosis (LB), the most common tick-borne disease in the Northern hemisphere caused by the bacterium *Borrelia burgdorferi* sensu lato. Genome-wide variants in LB patients—divided into a discovery and validation cohort—were compared to two healthy cohorts. Additionally, ex vivo monocyte-derived cytokine responses of peripheral blood mononuclear cells to several stimuli including *Borrelia burgdorferi* were performed in both LB patient and healthy control samples, as were stimulation experiments using mechanistic/mammalian target of rapamycin (mTOR) inhibitors. In addition, for LB patients, anti-*Borrelia* antibody responses were measured. Finally, in a subset of LB patients, gene expression was analysed using RNA-sequencing data from the ex vivo stimulation experiments.

**Results:**

We identified a previously unknown genetic variant, rs1061632, that was associated with enhanced LB susceptibility. This polymorphism was an eQTL for *KCTD20* and *ETV7* genes, and its major risk allele was associated with upregulation of the mTOR pathway and cytokine responses, and lower anti-*Borrelia* antibody production. In addition, we replicated the recently reported SCGB1D2 locus that was suggested to have a protective effect on *B. burgdorferi* infection, and associated this locus with higher *Borrelia burgdorferi* antibody indexes and lower IL-10 responses.

**Conclusions:**

Susceptibility for LB was associated with higher anti-inflammatory responses and reduced anti-*Borrelia* antibody production, which in turn may negatively impact bacterial clearance. These findings provide important insights into the immunogenetic susceptibility for LB and may guide future studies on development of preventive or therapeutic measures.

**Trial registration:**

The LymeProspect study was registered with the International Clinical Trials Registry Platform (NTR4998, registration date 2015–02-13).

**Supplementary Information:**

The online version contains supplementary material available at 10.1186/s12879-024-09217-z.

## Background

Lyme borreliosis (LB) is an infectious disease transmitted by ticks infected with *Borrelia burgdorferi* sensu lato (*B. burgdorferi* s.l.) spirochetes. Although infection occurs in a minority of individuals after a tick bite, in both Europe and the United States, the incidence of tick bites and LB has increased substantially in recent years [1–4]. The first and most common manifestation of LB is a red expanding skin lesion at the site of the tick bite termed erythema migrans (EM). Moreover, LB may induce a variety of signs and symptoms, either accompanying EM, or as part of disseminated disease manifestations, such as Lyme arthritis and Lyme neuroborreliosis [5]. Despite antimicrobial therapy, persistent symptoms are reported by a substantial minority of patients [6, 7].

The susceptibility to LB may depend on host factors, such as immunological responses [8]. Understanding genetic predisposition for LB may provide insights into the functional genomics of the disease and identify mechanistic targets for treatment and diagnosis, eventually contributing to development of personalized preventive or therapeutic strategies. A recent genome-wide association study (GWAS) investigated the contribution of genetic variation to LB susceptibility based on epidemiological and genetic data from the Finnish population [9]. In the current study, we established a large prospective study cohort of patients with physician-confirmed localized or disseminated LB in the Netherlands (the LymeProspect cohort), for which the prevalence and determinants of persistent symptoms after treatment for LB were assessed [6, 10]. The study protocol was published before [11]. As part of this study, patients were followed for one year through three-monthly questionnaires, and blood samples were collected at baseline and six weeks later. In this paper, we describe a GWAS conducted on this cohort, which we divided into a discovery and a validation cohort, and compared to two control cohorts of healthy individuals. We identified a genome-wide significant genetic variant that was associated with enhanced susceptibility of physician-confirmed LB. This variant was linked to increased *KCTD20* and *ETV7* gene expression, and subsequent enhancement of the mTOR pathway induced by *B. burgdorferi* s.l.. At the functional level, this was linked to altered cytokine responses and reduced anti-*Borrelia* antibody production. Our findings point towards an immunogenetic base for LB susceptibility.

## Methods

### Patient subjects

Between April 2015 and October 2018, adult patients with physician-confirmed EM or disseminated LB were included in the LymeProspect study within a maximum of seven days after initiation of antibiotic treatment for LB [11]. During a one year follow-up, online questionnaires were completed every three months, and blood samples were collected at baseline and six weeks later. A total of 1138 patients were included, of whom three did not complete any questionnaire, but did provide blood samples. These patients could not be included in the primary analyses of the study [6], but were part of the current analyses. Baseline characteristics are shown in Table 1. The study was approved by the medical ethics committee (METC) Noord-Holland (NL50227.094.14), and was conducted according to the principles of the Declaration of Helsinki. Written informed consent was obtained from all participants.

**Table 1:** Baseline characteristics of the discovery and validation cohorts of LB patients and controls

**Characteristic**	**Discovery**		**Validation**	
	**LB patients (*n* = 506)**	**Controls (*n* = 313)**	**LB patients (*n* = 557)**	**Controls (*n* = 441)**
Male sex—no (%)	197 (38.9%)	141 (43.3%)	241 (43.3%)	211 (43.1%)
Age (years)				
median [min, max]	54 [19, 82]	23 [18, 71]	56 [19, 87]	23 [18, 75]
mean (SD)	53.1 (13.3)	25.9 (10.6)	53.9 (11.8)	28.1 (13.5)
LB manifestation				
EM	465 (91.9%)	NA	556 (99.8%)	NA
Disseminated LB	41 (8.1%)		1 (0.2%)	
ACA	18		1	
LNB	10		NA	
Lyme arthritis	12		NA	
Other^a^	1		NA	

The discovery cohort was compared to the 300BCG cohort, the validation cohort was compared to the 500FG cohort. All but one patients with a disseminated LB manifestation were included in the discovery cohort, although numbers were few

*Abbreviations*: *ACA* acrodermatitis chronic atrophicans, *EM* erythema migrans, *LB* Lyme borreliosis, *LNB* Lyme neuroborreliosis

^a^Category ‘other’: early (sub)acute symptoms without erythema migrans

### Healthy controls

As a control, GWAS data from two independent Dutch population-based cohorts of healthy adults included at the Radboud university medical center (Nijmegen, the Netherlands) were available: the 300BCG cohort for the discovery cohort, and the 500FG cohort (see http://www.humanfunctionalgenomics.org) for the validation cohort [12, 13]. These cohorts were genotyped using the HumanOmniExpressExome-8 v1.0 (Illumina, San Diego, CA, USA) SNP array. The 300BCG cohort (the control for the discovery cohort) consisted of 326 healthy individuals, recruited between April 2017 and June 2018, and with an age ranging between 18 and 71 years, of whom for 313 genotype data was available. In the 500FG cohort (the control for the validation cohort), 534 healthy individuals between 18 and 75 years old were included between August 2013 and December 2014. From this cohort, cytokine production from PBMC stimulation experiments and RNAseq data were available as well. Both cohorts were approved by the ethics committee of the Radboud University Nijmegen (CMO Regio Arnhem-Nijmegen; 500FG cohort, NL42561.091.12; 300BCG cohort, NL58553.091.16), and were conducted in accordance with the Declaration of Helsinki. All healthy controls gave written informed consent.

### Buffy coats

For validation experiments, PBMCs from healthy volunteers were isolated from buffy coats (Sanquin Blood Bank, Nijmegen, the Netherlands). Blood samples were obtained after written informed consent, in accordance with the Declaration of Helsinki and after ethical approval from the ethics committee of the Radboud University Nijmegen (CMO Regio Arnhem-Nijmegen, NL32357.091.10).

#### *B. burgdorferi* serology

*B. burgdorferi* s.l. serology was performed at baseline and after six weeks, in a two-tier strategy, starting with a total Ig C6 Lyme ELISA (Immunetics/Oxford Immunotec, Oxford, United Kingdom), followed by IgM and IgG immunoblot analysis (*recom*Line Borrelia IgM and IgG, Mikrogen GmbH, Neuried, Germany) if equivocal or positive. All tests were performed in accordance with the manufacturer’s instructions.

### Whole blood and PBMC stimulation experiments

For all ex vivo cytokine experiments in the LB cohort (*n* = 1059), peripheral blood mononuclear cells (PBMCs) were isolated according to standardized protocols within 24 h after blood collection. Venous blood from two 10 ml heparine tubes was transferred to a 50 ml tube and diluted 1:1 in phosphate-buffered saline (PBS)-buffer (B. Braun, Melsungen, Germany). The PBMC fraction was obtained by density gradient centrifugation using Ficoll-Paque PLUS (GE Healthcare, Zeist, the Netherlands). Cells were washed three times in PBS, and resuspended to a concentration of 5 × 10^6^ cells/ml in RPMI 1640 (Dutch modification; Life Technologies, Breda, the Netherlands) supplemented with gentamicin (50 mg/L), pyruvate (1 mM), and L-glutamin (2 mM). Cells and stimuli were added 1:1 to each well of a round-bottom 96-wells cell culture plate (Greiner Bio-One, Kremsmünster, Austria). Live attenuated (i.e., thawn) *B. burgdorferi* s.l., consisting of a mix of three strains (*B. burgdorferi* sensu stricto (B31 strain, ATCC® 35210), *B. afzelii* (pKo isolate), and *B. garinii* (ATCC® 51383)), was used at various concentrations (5 × 10^3^ to 5 × 10^6^ spirochetes/mL). From September 2017 on, the lowest concentration of this *B. burgdorferi* s.l. mix was replaced by *B. afzelii* (pKo isolate) 5 × 10^5^/mL only. Spirochetes were cultured in Barbour-Stoenner-Kelley (BSK)-medium, supplemented with 6% rabbit serum. Next to the medium control, cells were also stimulated with lipopolysaccharide 100 ng/ml (purified lipopolysaccharide (LPS) from E. coli serotype 055:B5; Sigma-Aldrich, St. Louis, MO, USA), Pam3Cys 10 μg/ml (EMC microcollections, Tübingen, Germany), and heat killed *Candida albicans* (*C. albicans*) blastoconidia 1 × 10^6^/mL. In addition, heparinized whole blood was stimulated in 48-wells cell culture plates (Greiner Bio-One, Kremsmünster, Austria), by adding 400 μl of stimulus (medium, *B. burgdorferi* s.l. mix 7.5 × 10^6^, 2.5 × 10^6^, and 7.5 × 10^5^/mL, LPS, and Pam3Cys) to 100 μl of whole blood. For validation experiments on mTOR function, PBMCs (5 × 10^6^/mL) from 19 healthy blood donors were isolated (using buffy coats from Sanquin, Nijmegen, the Netherlands), and preincubated for one hour with either Rapamycin (LC Laboratories, Woburn, MA, USA) in two concentrations (10 or 100 nM), or Torin (Tocris, Abingdon, United Kingdom) in three concentrations (30, 100 or 300 nM). Cells were thereafter stimulated with the *B. burgdorferi* s.l. mix (1 × 10^6^ spirochetes/mL) or the *C. albicans*. After 24 h of incubation at 37°C and 5% CO_2_, supernatants from all PBMC and whole blood experiments were stored at -20°C until further assessment.

### Ex vivo cytokine measurements

Human IL-1β, IL-6, IL-10 and IL-1Ra in supernatants of the above mentioned ex vivo cytokine experiments were measured using commercially available ELISA kits (Sanquin Reagents, Amsterdam, the Netherlands, and R&D Systems, Minneapolis, MN, USA). All measurements were performed in accordance with the manufacturer’s instructions.

### DNA isolation and genotyping

The DNeasy® Blood & Tissue Kit (Qiagen, Hilden, Germany) was used to isolate DNA from EDTA whole blood samples, according to the manufacturer’s protocol. Isolated DNA was genotyped using the Infinium Global Screening Array MD v1.0 (GSA) BeadChip (Illumina, San Diego, CA, USA). In total, 1107 patient samples were genotyped in two batches (sequentially, in January 2018 and May 2019), dividing the total cohort in a discovery (*n* = 517) and validation cohort (*n* = 590).

### RNA sequencing

For a subset of physician-confirmed LB patients included through the clinical LB centers, stimulated PBMC pellets were stored in RNAprotect® Cell Reagent (Qiagen, Hilden, Germany) at -80°C, at baseline and after six weeks. In total, 12 EM patients en 11 ACA patients were selected for RNA sequencing, all of whom had baseline skin biopsy samples available from both the affected skin and contralaterally. All patients with EM and five with ACA had a positive *B. burgdorferi* s.l. culture or polymerase chain reaction (PCR), whereas the other six ACA patients had negative results for the skin biopsies, but positive *B. burgdorferi* serology (C6 IgM/IgG ELISA and IgG immunoblot). As a control, stimulated PBMC pellets (one time point) were available from ten age and gender matched healthy volunteers who reported no active LB or other infectious disease [14]. Total RNA was extracted from pellets stimulated with *B. burgdorferi* s.l. mix (5 × 10^6^/mL), *C. albicans*, and medium, using Qiagen’s AllPrep DNA/RNA/Protein Mini Kit according to the manufacturer’s instructions. Whole transcriptome analysis was performed using the GeneChip® Human Transciptome Array 2.0 (Affymetrix, Santa Clara, CA, USA).

### Genome data pre-imputation quality control

Quality control was performed using PLINK v1.9 [15]. During pre-imputation quality control, samples with a call rate ≤ 0.99, gender mismatch, and excessive difference in heterozygosity rate (> 3SD outside of the mean) were excluded for both the patient and control cohorts. Quality control per single-nucleotide polymorphism (SNP) was performed to exclude variants with a Hardy–Weinberg equilibrium *P* ≤ 0.001, a call rate ≤ 0.99 and a minor allele frequency (MAF) ≤ 0.01. Ethnic outliers, identified by merging multi-dimensional scaling plots of the samples with 1000 Genomes data, were excluded for further analysis, resulting in two cohorts of patients from western European ancestry only. From related individuals with identity by descent > 0.185, the sample with more missing data was removed.

### Cohort alignment, imputation and quality control

Since different genotyping platforms were used for the LB patient and control cohorts, only the overlapping variants between each case–control cohort pair could be used for imputation. The cohorts were aligned to the Haplotype Reference Consortium (HRC) 1.1 GRCh37 genotype before merging. Variants that could not be aligned were excluded. The alignment resulted in 88513 overlapping variants for the discovery cohort with the 300BCG cohort and 141780 variants for the validation cohort with the 500FG cohort. Subsequently, the discovery and validation cohorts were imputed separately on the Michigan imputation server, without using a R^2^ filter. The data were phased using Eagle v2.4 with the European population of HRC 1.1. 2016 hg 2019 as a reference panel. Post-imputation quality control excluded variants with a MAF < 0.01, an imputation quality score R^2^ < 0.5, or a Hardy–Weinberg-Equilibrium *P* < 10^–12^.

### Genome wide association study

Association analyses for GWAS of disease susceptibility was performed using PLINK v1.9 association function [15]. Association tests were performed separately for the discovery and validation cohort. Genome-wide significant SNPs (*p* < 5.0 × 10^–8^) in the discovery cohort were inspected in the validation cohort and considered candidate SNPs if validation *p*-value was below the suggestive-significant threshold (*p* < 1.0^–5^). Quantitative trait loci (QTL) were mapped to the average symptom score using a linear model with age, gender, genotyping batch and institute of collection as covariates, for which MatrixEQTL was used [16]. Both QTL and GWAS were performed separately for the discovery and validation cohort. To compare both cohort results, SNPs with nominal significance (*p* < 0.05) and shared direction between cohorts were considered. Direction was established as the sign of log odds ratio on association testing and the sign of beta values in QTL mapping. Combined *p*-values of both cohorts were calculated using weighted z-score analysis [17].

### Annotation of candidate SNPs

Candidate SNPs, as stated in the previous section, were annotated based on public cis eQTL [18], regulomeDB annotations [19], intersection with existing GWAS catalogs by phenoscanner [20, 21], and functional annotation of SNP2GENE in FUMA [22]. SNP effect on cytokine production was inferred based on the published data from the 500FG (validation control) cohort. Comparisons on the effect of allele dosage on cytokine expression were performed by using a linear model including age, gender and cell count as covariates [12]. Inferred transcription factor interactions were tested using available RNAseq data from the 500FG (validation control) cohort. Testing was limited to the expression and interaction of the identified transcription factor and its target gene. Spearman correlation and Wilcoxon rank-sum testing were performed.

### RNA expression analysis

RNAseq reads were quality controlled, preprocessed and aligned to the reference genome build resulting in a count matrix. Raw read counts were normalized with DESeq2 [23] and the distribution of expression levels in the genes of interest among conditions was tested using Wilcoxon rank-sum.

### Statistics

Other statistical details of experiments can be found in the figure legends, including definition of significance. For all analyses, R version 4.1.3. was used.

## Results

### Overview of patients and controls

**Fig. 1:**
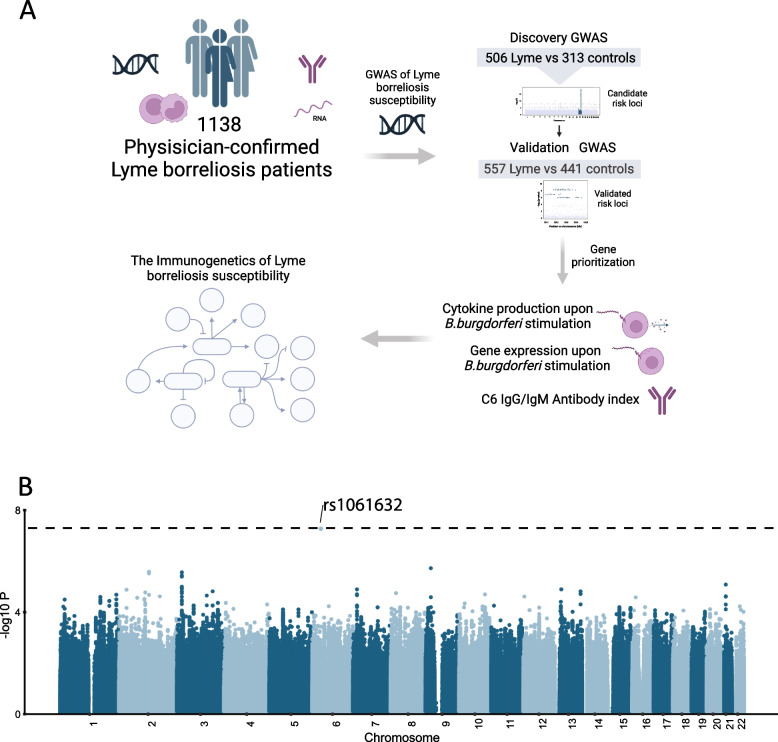
Identification of the rs1061632 variant to be associated with LB susceptibility. **A** Cohorts overview. 1,107 DNA samples from LB patients were available for quality check and imputation, leaving a discovery cohort (*n* = 506) and a validation cohort (*n* = 557). Furthermore, cytokine production upon stimulation experiments on PBMCs and whole blood, as well as antibody responses were assessed at baseline and six weeks later. **B** Manhattan plot of genome wide significant variants associated with susceptibility to LB in the discovery cohort. Chromosomal location is displayed on the x-axis, and -log_10_ *p*-values of SNPs on the y-axis. The dashed horizontal line indicates the genome-wide threshold for association (*p* = 5 × 10^–8^). The significant variant identified in the discovery cohort (rs1061632, *p* = 5.38 × 10^–8^, OR = 0.44), was significant in the validation cohort as well (*p* = 1.15 × 10^–5^, OR = 0.54)

For 1,107 out of 1,138 LB patients who were included in the LymeProspect study, DNA was available and consecutively genotyped in two batches using Illumina genotyping array (Fig. 1a). After imputation, quality control and cohort alignment, the discovery cohort consisted of 506 LB patients, compared with a cohort of 313 healthy controls. The validation cohort included 557 LB patients, compared with 441 controls. All individuals were of Western European ancestry. Genotype information was available for 6,116,499 and 7,378,650 variants, respectively. Out of the 1,063 LB patients in the discovery and validation cohorts, 1,021 (96.1%) were diagnosed with EM and 42 (3.9%) with disseminated LB. Baseline characteristics of all cohorts are shown in Table 1. The mean age was significantly lower for control cohorts than for LB patients, which was adjusted for in the cytokine analyses.

### Genome-wide association studies identify variant rs1061632 to be significantly associated with LB, and the *KCTD20* and *ETV7* genes are prioritized as the potential causal genes

**Fig. 2:**
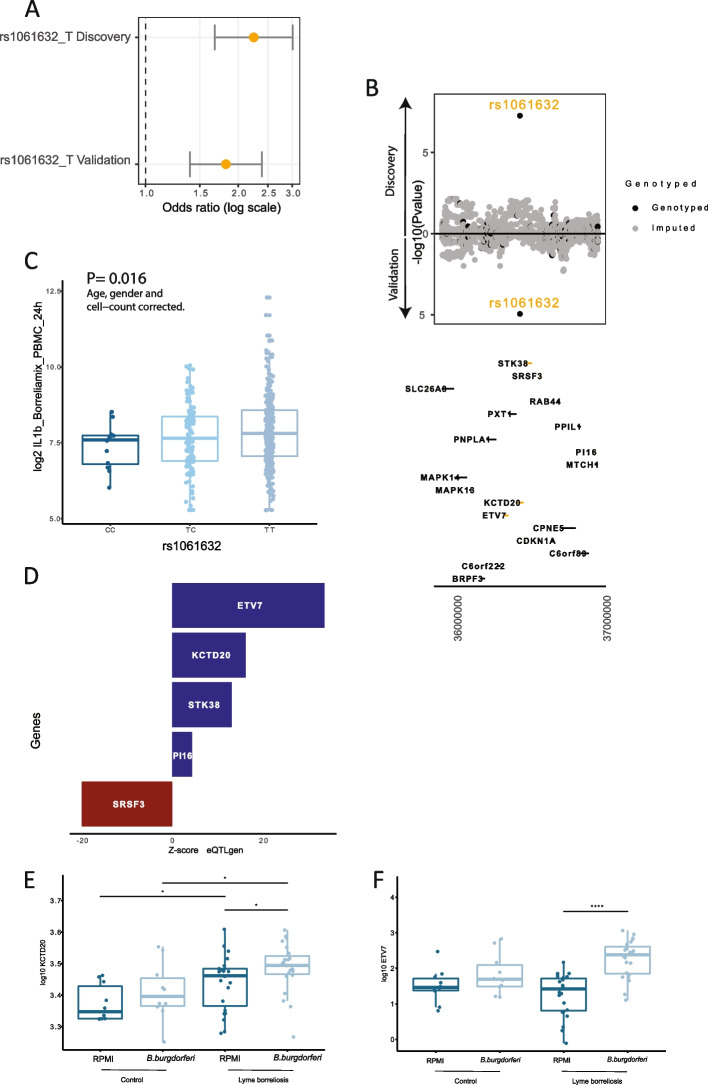
*ETV7* and *KCTD20* were identified to be affected by the rs1061632 variant. **A** The major allele (T) was associated with a higher odds ratio for LB in both discovery and validation cohorts. **B** Regional association plot for rs1061632. Chromosomal position is displayed on the x-axis, in the lower part of the figure. -log_10_ *p*-values of SNPs are on the y-axis; positive values indicate association in the discovery cohort and negative values in the validation cohort. Each dot represents a variant, colored by the source, either genotyped or imputed. **C** The major allele (T) was associated with higher IL-1β production upon stimulation of PBMCs with *B. burgdorferi* s.l. for 24 h in the validation control cohort (cQTL). Together with increased IL-1β production upon PBMC stimulation for 24 h with *M. tuberculosis*, and IL-6 production upon whole blood stimulation for 48 h with *S. aureus*, those were the significant associations among a wide range of stimuli and cytokines [12]. *P*-value is age, gender and cell count corrected. **D** eQTL mapped genes to rs1061632. Values presented are extracted from the public cis eQTL results in whole blood samples of healthy individuals of the eQTLgen consortium. Z score as available for the risk allele (T). Only significant genes are reported. **E** Higher expression of *KCTD20* was observed for the subset of patients from our LB cohort for whom RNA sequencing was performed (at baseline and after six weeks), compared to the healthy controls (at one time point), both in unstimulated and stimulated conditions. Stimulation with *B. burgdorferi* resulted in higher *KCTD20* expression than in unstimulated conditions. Pairwise comparison *p*-values represent the result of Wilcoxon rank sum test. **F** Higher expression of *ETV7* was observed for the subset of patients from our LB cohort for whom RNA sequencing was performed, when stimulated with *B. burgdorferi* s.l. compared to unstimulated conditions. Values reported were normalized by DESeq2. Pairwise comparison *p*-values represent the result of Wilcoxon rank sum test. * *p* < 0.05, ** *p* < 0.01, *** *p* < 0.001, **** *p* < 0.0001

A genome-wide association study comparing the genetic makeup of physician-confirmed LB patients and healthy controls revealed one independent locus significantly (*p* ≤ 5 × 10^–8^) associated with LB in the discovery cohort, which could be confirmed in the validation cohort (Fig. 1b, Supplementary Fig. 1). This single nucleotide polymorphism (SNP), rs1061632, was associated with LB with a *p*-value of 5.38 × 10^–8^ and an odds ratio of 0.44 in the discovery cohort, and with a *p*-value of 1.15 × 10^–5^ and an odds ratio of 0.54 in the validation cohort (Fig. 2a). Its minor allele (C) was less often present in LB patients (allele frequency 0.102, compared to 0.208 and 0.177 in the control cohorts), thus the major allele (T) was correlated with a higher risk for LB, and the minor allele (C) was associated with a 56% (95%-CI 40–66%) reduction in risk of developing LB compared to the major allele (T). The variant is located on chromosome 6 in a DNA binding motif specific for the interferon regulated factor 7 (IRF7; ENCODE, ENCSR692TXU) [24], within the 3’ UTR region of the *KCTD20* (potassium channel tetramerization domain containing 20) gene (Fig. 2b). The potential functional effects of rs1061632 on cytokine production (cytokine quantitative trait locus, cQTL) were explored using available data from the validation control cohort (500FG) [12]. The major allele (T) was significantly associated with higher interleukin (IL)-1β production upon stimulation of peripheral blood mononuclear cells (PBMCs) with *B. burgdorferi* s.l. (Fig. 2c). To identify *cis*-genes potentially affected by rs1061632, publicly available expression quantitative trait loci (eQTLs) as from the Genotype-Tissue Expression (GTEx) project [25], the eQTLGen Consortium [26], and BIOSQTL databases [18] were used. This eQTL mapping at rs1061632 showed significant upregulation of four genes, particularly *ETV7* (ETS Variant Transcription Factor 7) and *KCTD20*, in whole blood samples of healthy individuals harboring the major allele (Fig. 2d). The genes in this locus (within a 500 kb window of rs1061632) were further prioritized by testing if they were differentially expressed between patients and controls in a publicly available PBMC transcriptome dataset [27]. In LB patients included in this dataset, amongst several up- and downregulated genes (Supplementary Fig. 2), the *ETV7* and *KTCD20* genes were upregulated in the acute phase of infection, before start of antibiotic treatment. In transcriptomic data from PBMC stimulation experiments in a subset of LB patients from our cohort, *KCTD20* expression was upregulated compared with healthy controls, both in stimulated and unstimulated conditions (Fig. 2e). Moreover, expression of *ETV7* and *KCTD20* was significantly increased in PBMCs stimulated with *B. burgdorferi* s.l. compared with unstimulated conditions (Fig. 2e,f). Taken together, the association of the rs1061632 major (T) allele with higher LB susceptibility, and with higher cytokine response upon *B. burgdorferi* s.l. stimulation, affecting *ETV7* and *KTCD20* genes in whole blood, both of which were upregulated in ex vivo experiments and in LB patients, suggested a functional role for *ETV7* and *KTCD20* in the human host defense against *B. burgdorferi* s.l.

### *ETV*7* and KCTD20 *are involved in the Akt-mTOR pathways in the host immune response against *B. burgdorferi* s.l., both directly and through IRF7

**Fig. 3:**
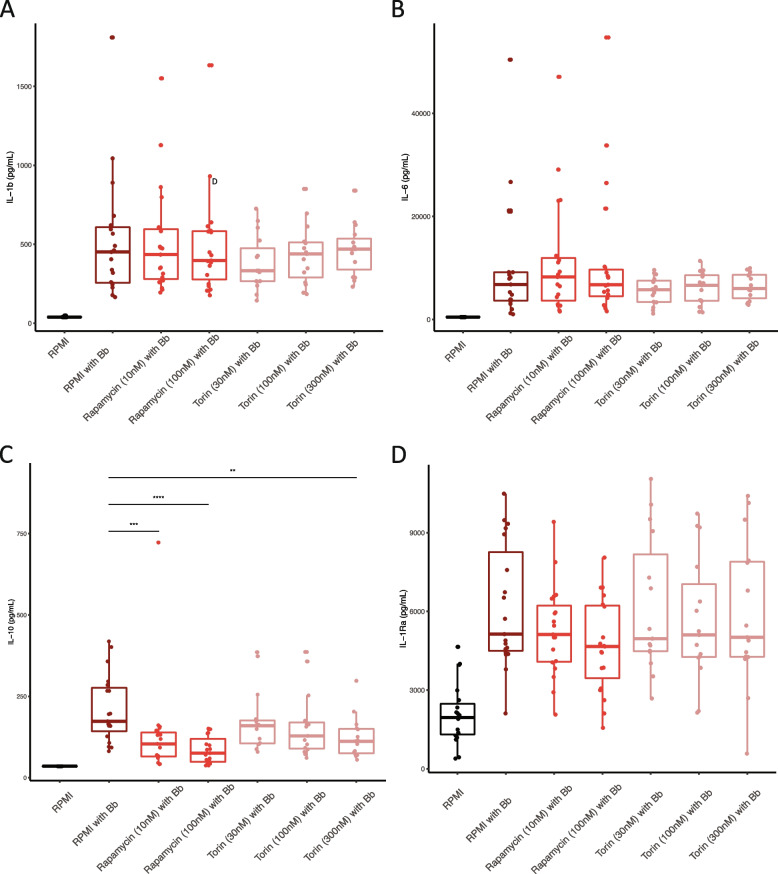
The rs1061632 variant has functional associations with the mTOR pathway. **A**-**D** The role of the Akt-mTOR pathway in *B. burgdorferi* s.l. recognition was tested in PBMC stimulation experiments. PBMCs from healthy donors (buffy coats) were stimulated with *B. burgdorferi* s.l. in the presence or absence of mTOR inhibitors in various concentrations. Concentrations of (**A**) IL-1β, (**B**) IL-6, (**C**) IL-10, and (**D**) IL-1Ra were determined after 24 h of stimulation. IL-10 concentrations were significantly decreased in a dose-dependent manner when mTOR was inhibited. This situation reverses the suggested effect of the rs1061632 major allele. *P*-values were calculated using Wilcoxon rank-sum test, ** *p* < 0.01, *** *p* < 0.001, ****p < 0.0001

**Fig. 4:**
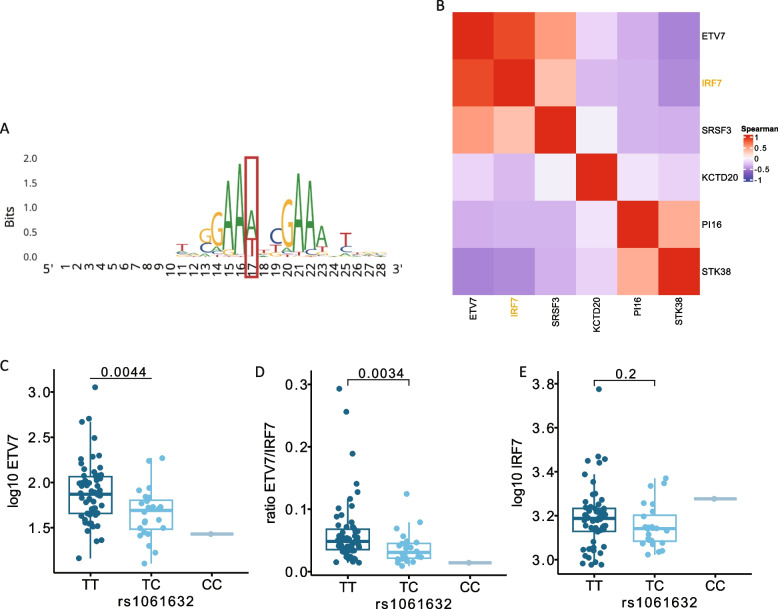
The rs1061632 variant alters ETV7 expression through the regulation of IRF7 binding. **A** IRF7 motif extracted from the RegulomeDB. The red box indicates the nucleotide altered by rs1061632. **B** Correlation of the genes affected by rs1061632 with IRF7. Color indicates the Spearman correlation of gene expression in PBMCs extracted from 100 healthy controls from the validation control (500FG) cohort. **C**-**E** Effect of rs1061632 on (**C**) ETV7 expression, (**D**) ETV7/IRF7 ratio, and (**E**) IRF7 expression, as extracted from the RNAseq data from the validation control (500FG) cohort. Gene expression counts were normalized using DEseq2 and log10 transformed. *P*-values were calculated using Wilcoxon rank-sum test

**Fig. 5:**
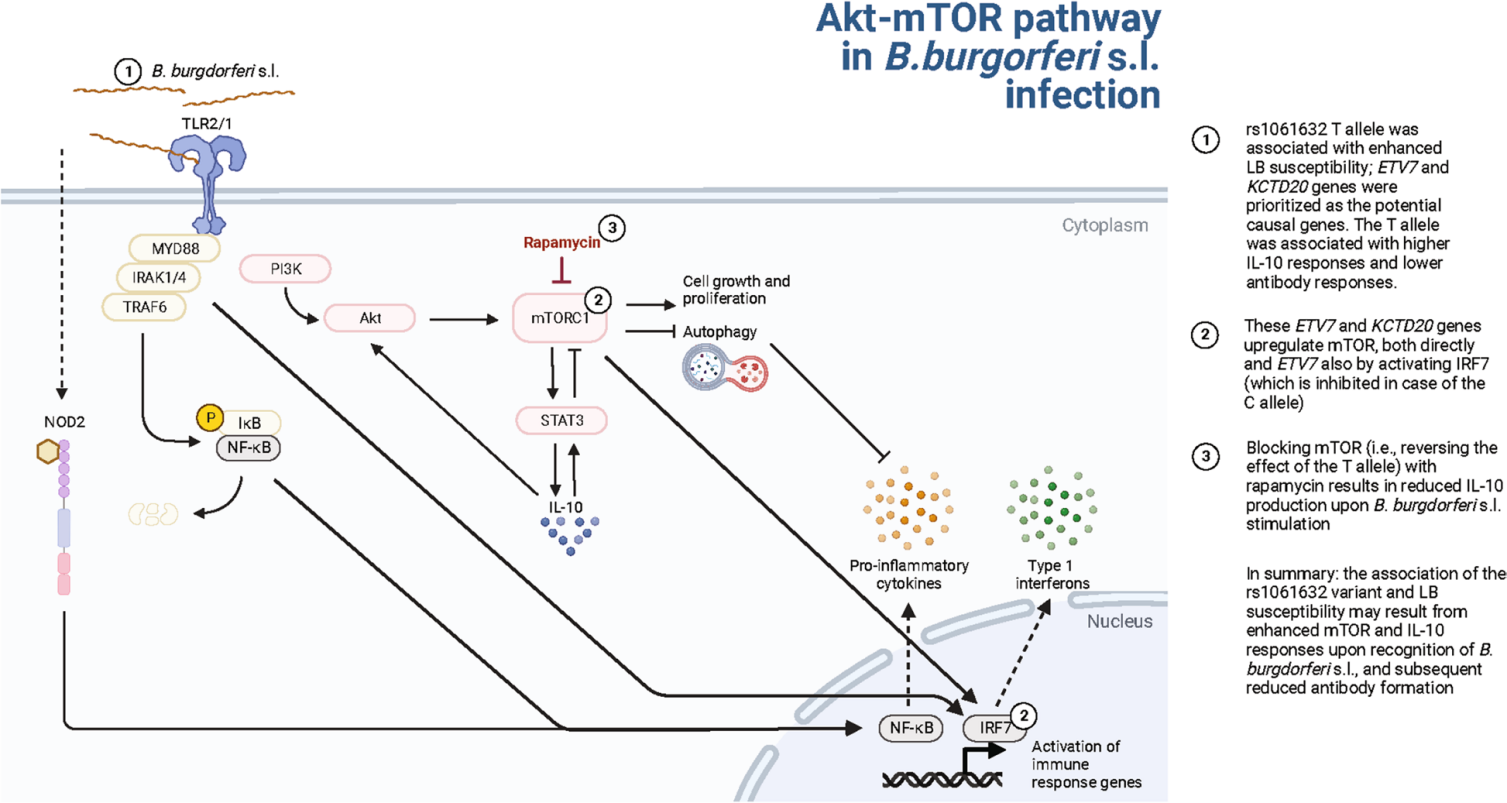
Proposed mechanisms of LB susceptibility in rs1061632 major variant. Upon recognition of *B. burgdorferi* s.l.-specific ligands by pattern recognition receptors, various signaling cascades are activated in the host immune cells, of which PI3K-Akt-mTOR is one. In addition to proinflammatory cytokines via the MYD88-NF-κB pathway, anti-inflammatory IL-10 is produced, particularly upon mTOR activation. Rapamycin is a mTOR complex 1 inhibitor. The association between the rs1061632 major allele and increased LB susceptibility may relate to enhanced mTOR and IL-10 responses and subsequent reduced antibody formation

The mechanistic/mammalian target of rapamycin (mTOR) is a serine/threonine kinase that plays a central role in regulating cell metabolism, proliferation, survival and autophagy. mTOR is also a regulator of immune cells, thereby influencing the control of infection. Upon various intracellular and extracellular signals, including Toll-like receptor signaling and cytokines, intracellular mTOR complex 1 and mTOR complex 2 are activated through the PI3K-Akt pathway [28]. This pathway has been described to be involved in the host immune response against *B. burgdorferi* s.l. [29, 30] *ETV7* is a protein coding gene that is interferon-stimulating and has been described to create a complex with mTOR, thereby playing a role in inducing rapamycin resistence [31]. *KCTD20* is a protein coding gene, promoting the phosphorylation of Akt, thus participating in the Akt-mTOR signaling cascade [32]. While the risk allele of the rs1061632 was linked to higher cytokine production (Fig. 2c), validation experiments in samples from healthy donors showed that inhibition of mTOR in *B. burgdorferi* s.l. stimulated PBMCs resulted in dose-dependent reduction of anti-inflammatory cytokines, in particular IL-10 (Fig. 3). Furthermore, *ETV7* was positively correlated to interferon regulated factor 7 (IRF7) in transcriptome data from our physician-confirmed LB patients (*r* = 0.85, *p* < 2.2^–16^) (Fig. 4b), and its nucleotide motif was altered by rs1061632 (Fig. 4a). IRF7 is an important regulator of type 1 interferons (IFN-α and IFN-β) production, activated upon pathogen recognition via Toll-like receptors (TLR)2, -3, -7 and -9 [33]. Interestingly, the PI3K-Akt-mTOR was reported to be critical for the activation of IRF7 in dendritic cells [34]. The effect of the rs1061632 variant on the expression of *ETV7* and IRF7 was assessed in RNAseq data from 100 healthy individuals of the validation control cohort (500FG) (Fig. 4c-e) [35].To test in silico the hypothesized activating effect of IRF7 on *ETV7* expression, the ratio of two transcripts was composed, and showing a higher IRF7/*ETV7* ratio (*p* < 0.005) in individuals with the major allele (T) (Fig. 4d). This aligns with the fact that the C allele of the rs1061632, that is related to a lower frequency of LB, alters the binding motif (ENCODE, ENCSR692TXU) [24], potentially leading to diminished binding properties of IRF7 to the binding site, resulting in lower activation of *ETV7*. Altogether, we hypothesize that the major allele T of the rs1061632 variant increases LB susceptibility by its association with higher *ETV7* and *KTCD20* expression, which functionally affects host immune (cytokine) responses by upregulating the Akt-mTOR pathway upon *B. burgdorferi* s.l. recognition, both directly and via IRF7 (Fig. 5).

### Functional effects of the SCGB1D2 variant, recently associated with increased susceptibility to LB: higher antibody responses and lower IL-10 production upon *B. burgdorferi* s.l.

**Fig. 6:**
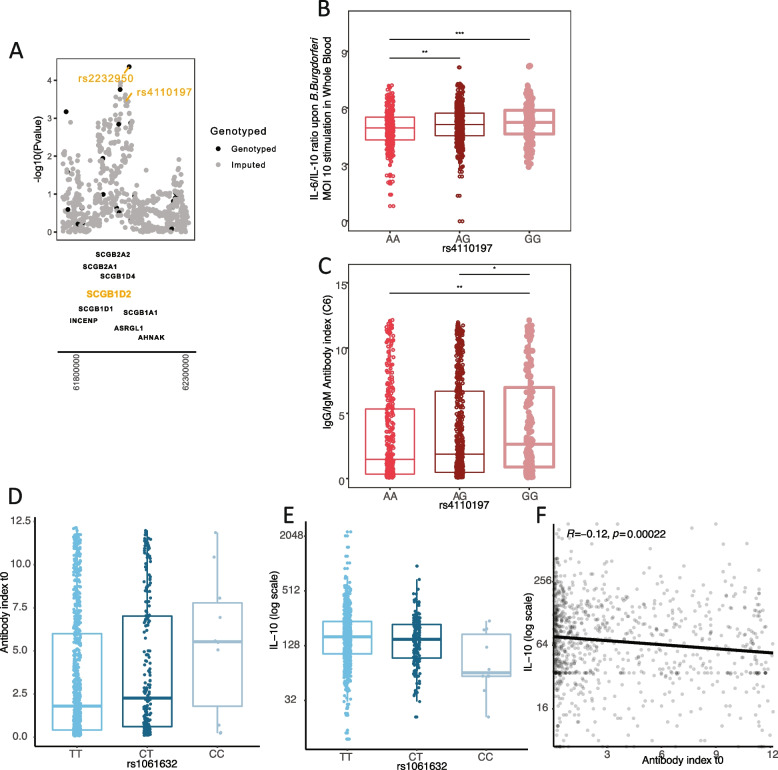
LB susceptibility variants were associated with cytokine and antibody production. **A** A suggestive hit (*p* < 1.0^–4^) was observed for the missense variant rs2232950 and for rs4110197 after meta-analyzing the discovery and validation cohort. **B** Boxplots showing the relevance of LB susceptibility variant rs4110197 for baseline cytokine production in our physician-confirmed LB patients. **C** Boxplots showing the relevance of LB susceptibility variant rs4110197 for baseline antibody production in our physician-confirmed LB patients. **D** Association between rs1061632 genotypes and baseline *B. burgdorferi* s.l. antibody indexes (C6 IgM/IgG ELISA) in our physician-confirmed LB patients, *p* = 0.01 age, sex, batch, and institute corrected (linear model). **E** Association between rs1061632 genotypes and IL-10 production upon stimulation of whole blood samples with *B. burgdorferi* s.l. mix (MOI 10) at baseline in our physician-confirmed LB patients, *p* = 0.04 age, sex, batch, and institute corrected (linear model). **F** Negative Pearson correlation between *B. burgdorferi* s.l. antibody indexes (C6 IgM/IgG ELISA) and IL-10 production upon stimulation of PBMCs with *B. burgdorferi* s.l. 10^5^/ml in our physician-confirmed LB patients at baseline

Recently, a missense variant at the gene encoding for the Secretoglobin family 1D member 2 (SCGB1D2) protein was linked to LB in a large Finnish database (FinnGen), where genetic data was linked to LB diagnosis as recorded by International Classification of Diseases codes [9], This protein is primarily expressed in the skin and sweat glands, and was found to inhibit growth of *B. burgdorferi* s.l. in vitro, suggesting that SCGB1D2 may have a protective effect on *B. burgdorferi* s.l. skin infection. When we meta-analyzed our discovery and validation cohorts, we were able to replicate the association of the sentinel variant (rs4110197; *p* < 1 × 10^–4^) with physician-confirmed LB (Fig. 6a). Interestingly, the causal candidate variant (missense variant rs2232950) from this locus reported by Finnish study showed a stronger association in our meta-analysis result (*p* = 4.381e-05), compared to the top variant in this locus (rs4110197; *p* < 1 × 10^–4^). Similar to the top SNP in our study (r1061632), rs4110197 was significantly associated with immunological parameters, i.e. higher *B. burgdorferi* s.l. antibody indexes (as measured by C6 IgG/IgM ELISA), lower IL-10 responses, and higher IL-6 responses (Fig. 6b,c) in our physician-confirmed LB cohorts. To sum up, our GWAS result replicated the recently reported genetic, protective effect of the SCGB1D2 locus for LB susceptibility and showed potential diminishing effects on anti-inflammatory responses, which aligns with the identified GWAS locus (rs1061632) from our study.

### rs1061632 LB risk allele was associated with higher IL-10 and lower antibody responses

We next assessed the relation between LB susceptibility, IL-10 responses, and antibody production in our physician-confirmed LB cohorts. As we expected, we observed that the rs1061632 risk allele T was associated with lower baseline antibody indexes (*p* = 0.010) and with higher IL-10 responses upon *B. burgdorferi* s.l. recognition, particularly in whole blood experiments (*p* = 0.040) (Fig. 6d,e). Also, using Pearson’s correlation we found a correlation between higher antibody responses and lower IL-10 production (R = -0.12, *p* = 0.00022) (Fig. 6f). Therefore, we put forward that immunogenetic variation leading to enhanced IL-10 responses and reduced antibody production upon *B. burgdorferi* s.l. recognition plays an important role in LB susceptibility.

## Discussion

In this prospective cohort study of 1,063 patients with Lyme borreliosis (LB) and two control cohorts, we identified the rs1061632 major allele (T) variant as a susceptibility variant for LB. The variant was linked to upregulated expression of the *ETV7* and *KCTD20* genes, both of which share a functional link with the mTOR signaling network [31, 32] and resulted in induced pro-inflammatory cytokine responses upon *B. burgdorferi* s.l. stimulation. The association of LB susceptibility with the mTOR pathway was endorsed by the finding of an activating effect of IRF7 on *ETV7*, the previously described association of the minor allele with diminished IRF7 function, and the known role for mTOR in IRF7 activation [34]. As a functional result of upregulated mTOR activity, the risk variant was associated with increased cytokine production (IL-1β and IL-6) upon *B. burgdorferi* s.l. stimulation of PBMCs from healthy donors. When blocking mTOR activity, regardless of genotype, cytokine production was dose-dependently suppressed, an effect that was particularly observed for IL-10. In addition, we were able to confirm the recent finding that the SCGB1D2 protein is associated with LB susceptibility and showed functional associations with upregulated IL-6 and antibody responses, and, again, diminished IL-10 responses in LB patients.

Various key regulatory cell processes have been assigned to mTOR, which comprises two distinct complexes (mTOR complex 1 and 2). Dysfunction has been linked to human disease, particularly to cancers and inflammatory diseases. During infection, pathogens may target mTOR to hijack or disturb host immune processes [36]. Host-immune cells are activated upon recognition of pathogen-associated molecular patterns (PAMPs) by pattern recognition receptors (PRRs), including TLRs. Amongst others, extracellular TLR2 (and heterodimers with TLR1 and TLR6) and intracellular nucleotide-binding oligomerization domain-containing protein (NOD)2 are particularly important for the recognition of *B. burgdorferi* s.l. spirochetes [37, 38], initiating intracellular cascades that result in inflammatory responses. The PI3K-Akt-mTOR pathway is one of these signal transduction cascades, resulting in activation of mTORC1 and subsequent immune responses. Activation of mTOR leads to a network of responses and feedback loops, that sometimes appear to be constrasting [39]. Pathogens may affect the complex mTOR activation, or bypass its activity [36]. The exact interaction between *B. burgdorferi* s.l. recognition and mTOR activation is largely unknown, but the Akt-mTOR pathway has previously been reported to affect cytokine and ROS production in PBMCs stimulated with *B. burgdorferi* s.l. [29, 30] In the current study, we identified a genetic variant associated with increased susceptibility to LB. This variant regulated expression of the *ETV7* and *KCTD20* genes, and we hypothesized that this resulted in enhanced LB susceptibility through upregulation of the mTOR pathway upon *B. burgdorferi* s.l. recognition. The functional association between *ETV7* and *KCTD20* with the mTOR pathway is known from literature [31, 32]. Additionally, the rs1061632 C allele (i.e., lower LB frequency) was linked to diminished IRF7 binding potential, which is a downstream factor of mTOR [34], and to lower *ETV7* activation in our cohorts. Thus, our findings suggest that LB susceptibility in individuals with the major rs1061632 allele results from enhanced *ETV7* and *KCTD20* gene expression and subsequent activation of the Akt-mTOR pathway.

When inhibiting mTOR complex 1 in PBMC stimulation experiments in healthy donor samples, we observed reduced IL-10 production upon *B. burgdorferi* s.l. stimulation. IL-10 is an anti-inflammatory cytokine with a dampening effect on inflammatory processes, thereby minimizing tissue damage. A negative feedback loop has been suggested, in which mTOR activation induces IL-10 production, and IL-10 suppresses mTOR activation via STAT3 (Fig. 5). The reduced IL-10 production by PBMCs stimulated with *B. burgdorferi* s.l. after blocking the mTOR pathway aligns with these findings, and with our previously published findings that *B. burgdorferi* s.l.-induced production of IL-22, an IL-10 family member, was reduced upon mTOR inhibition [30]. In addition, in experiments with *Leishmania donovani*, a pathogen that is also recognized by TLR2, mTOR blocking also resulted in decreased IL-10 production [40]. Generally, inappropriate IL-10 responses have been associated with chronic infection [41]. In patients treated for Lyme arthritis, ongoing inflammation and autoimmunity responses were associated with high pro-inflammatory IFN-γ concentrations in combination with low IL-10 levels [42]. Altogether, these data point to an association between enhanced mTOR activation and higher IL-10 responses in LB, which may result in dampening of the pro-inflammatory immune responses upon *B. burgdorferi* s.l. recognition.

Further studies are needed to unravel the exact mechanism by which upregulated mTOR activation affects the host immune response to *B. burgdorferi* s.l., and thus LB susceptibility. When mTOR is active and is thus promoting cell growth and proliferation, autophagy, an important intracellular process to degrade unnecessary or damaged cell components, is inhibited [43]. Although basal levels of autophagy are mTOR independent, enhanced mTOR activation may result in a diminished level of autophagy [36]. Subsequently, pro-inflammatory responses may be induced [44], which corresponds with the finding of higher IL-1β responses upon *B. burgdorferi* s.l. stimulation in the variant’s major allele (Figs. 2c and 5), Other processes that could be involved include suppressed ROS-production [29, 45], and inhibition of Th1 cell cytokine responses (e.g., IFN-γ and TNF-α) [46], possibly through induced IL-10 production. Moreover, bacterial clearance may be affected, since murine studies suggest that an IL-10 deficit is related to increased disease severity on the one hand, and reduced levels of *B. burgdorferi* s.l. spirochetes and higher antibody levels on the other hand [45, 47]. Furthermore, one could hypothesize that increased cytokine production upon enhanced mTOR activity results in more symptomatic, thus manifest, LB, rather than in a higher LB infection rate. Also, we cannot rule out that the suggested association of enhanced mTOR activity with LB susceptibility is age-dependent to some extent. Our patient cohorts were aged 54–56 years at median, while the median age in the control cohorts was 23 years. Epidemiological studies have shown a bimodal age distribution of LB, with the highest incidence at childhood (5–15 years) and adult age (45–55 years) [48]. Although this distribution may relate to behavioral factors, immunological changes over time (a process that is referred to as immunosenescence) could play a role as well. Finally, we here report for the first time an association of the rs1061632 variant with susceptibility to an infectious disease, and specifically to LB. Future studies will have to assess *B. burgdorferi* s.l. specificity.

In addition to the finding that the mTOR pathway may have a role in LB susceptibility, the SCGB1D2 protein was recently described to affect LB susceptibility [9]. Being expressed in skin and sweat glands, this protein was suggested to have a direct *B. burgdorferi* s.l. killing effect, based on epidemiological data combined with functional experiments. In our prospective cohort of physician-confirmed LB patients, we were able to replicate the genetic association of SCGB1D2 polymorphisms with LB, and to functionally validate it. The protective variant related to lower IL-10, higher IL-6 and higher antibody responses upon *B. burgdorferi* s.l. exposure in the LB patients. Antibody responses, as measured with C6 IgM/IgG ELISA, were positive in 59% patients at baseline, which is in line with previous literature [49]. Our findings support the positive association between enhanced LB susceptibility and IL-10 responses. In addition, our findings confirm a negative association for IL-10 with *B. burgdorferi* s.l. antibody responses, which may eventually result in impaired bacterial clearance.

One of the limitations of the current study lies in the imputation of variants in high linkage disequilibrium (LD) with rs1061632. Only rs1061632 was associated with the disease, while none of the variants in high LD with rs1061632 were found to be significanly associated with the disease. This could be an effect of imputation, as rs1061632 was directly measured by the genotyping array and the rest of the variants was imputed. Furthermore, this study was limited by the parameters that were available for functional analyses. We report important associations for mTOR and IL-10 in LB, while realizing that immune pathways are usually complex by their interactions with other pathways and by feedback mechanisms. Other pathway components may also have had an important role in the hypothesized mechanisms, e.g., interaction with IL-12 and ERK1/2 [46, 50, 51]. Moreover, since the majority of the LB cohort in this study were patients with erythema migrans, the reported variant could mainly be associated with erythema migrans specific characteristics. We suggest that future studies include a higher number of patients with disseminated LB manifestations to take this into account. Finally, our findings provide insight into immunogenetic regulation of LB susceptibility, and further studies are needed to confirm causality.

## Conclusions

This genome-wide association study of physician-confirmed LB patients in a prospective cohort demonstrates that in individuals with the rs1061632 major allele, activated mTOR upon *B. burgdorferi* s.l. results in upregulated IL-10 responses, and have a genetical base in induced expression of *ETV7* and *KCTD20* genes. Our data build on previous findings and suggest that LB susceptibility is enhanced in patients with higher anti-inflammatory responses, influencing innate and adaptive immune responses, as reflected by reduced specific anti-*Borrelia* antibody production. This might negatively impact bacterial clearance. The exact underlying mechanism warrants further study and may direct future studies on preventive measures.

### Supplementary Information

**Supplementary Material 1. **

## Data Availability

The datasets used and/or analysed during the current study are available from the corresponding author on reasonable request. Original codes used for the analyses are available at GitHub (https://github.com/CiiM-Bioinformatics-group/LymeProspect_GWAS) and is publicly available as of the date of publication. Any additional information required to reanalyze the data reported in this paper is available from the corresponding author upon request.

## Abbreviations

ACA: Acrodermatitis chronica atrophicans
B. burgdorferi: Borrelia burgdorferi
C. albicans: Candida albicans
cQTL: Cytokine Quantitative Trait Locus
EM: Erythema migrans
eQTL: Expression Quantitative Trait Locus
GWAS: Genome-wide association study
IFN: Interferon
Ig: Immunoglobulin
IL: Interleukin
LB: Lyme borreliosis
LD: Linkage disequilibrium
LNB: Lyme neuroborreliosis
LPS: Lipopolysaccharide
MAF: Minor allele frequency
MOI: Multiplicity of infection
mTOR: Mechanistic or mammalian target of rapamycin
OR: Odd ratio
PAMP: Pathogen-associated molecular pattern
PBMC: Peripheral blood mononuclear cell
PCR: Polymerase chain reaction
PRR: Pattern recognition receptor
QTL: Quantitative Trait Locus
ROS: Reactive oxygen species SD Standard deviation
s.l.: Sensu lato
s.s.: Sensu stricto
SNP: Single-nucleotide polymorphism
TLR: Toll-like receptor
TNF: Tumornecrosisfactor
