# Supplementary material for: Genome-wide analyses in Lyme borreliosis: identification of a genetic variant associated with disease susceptibility and its immunological implications
Source: BMC Infect Dis. 2024 Mar 21;24:337. doi: 10.1186/s12879-024-09217-z (PMC10956190; doi:10.1186/s12879-024-09217-z)
Supplement: Supplementary file 1 — Supplementary Material 1. [file 12879_2024_9217_MOESM1_ESM.pdf]

Additional file 1

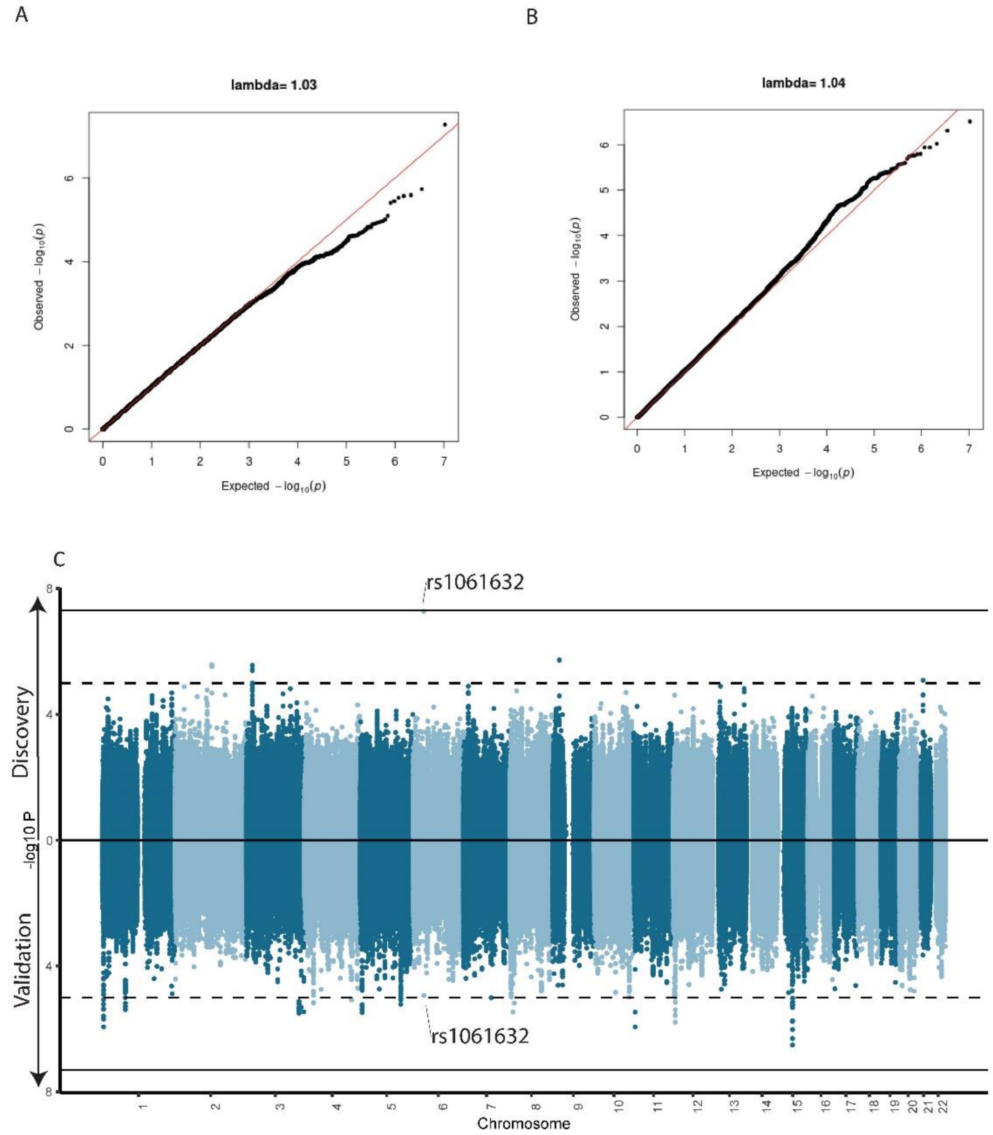

Supplementary figure 1. Quantile-Quantile (QQ) and Manhattan plots for discovery and validation cohorts.

(A-B) QQ plots for the (A) discovery and (B) validation cohorts. Expected -log10 p-values (x-axis) are compared to observed values (y-axis). Lambda values indicate genomic inflation factor, as the ratio between the median of the observed distribution and the median of the expected distribution, suggestion no population stratification.

(C) Manhattan plot of genome wide significant variants associated with susceptibility to LB in the discovery cohort. Chromosomal location is displayed on the x-axis, and -$\log_{10}$ p-values of SNPs on the

y-axis. The continuous horizontal line indicates the genome-wide threshold for association ($p=5x10^{-8}$) and the dashed one denotes the suggestive threshold ($p=1.00x10^{-5}$). The significant variant identified in the discovery cohort (rs1061632, $p=5.38x10^{-8}$, OR=0.44), was significant in the validation cohort as well ($p=1.15x10^{-5}$, OR=0.54).

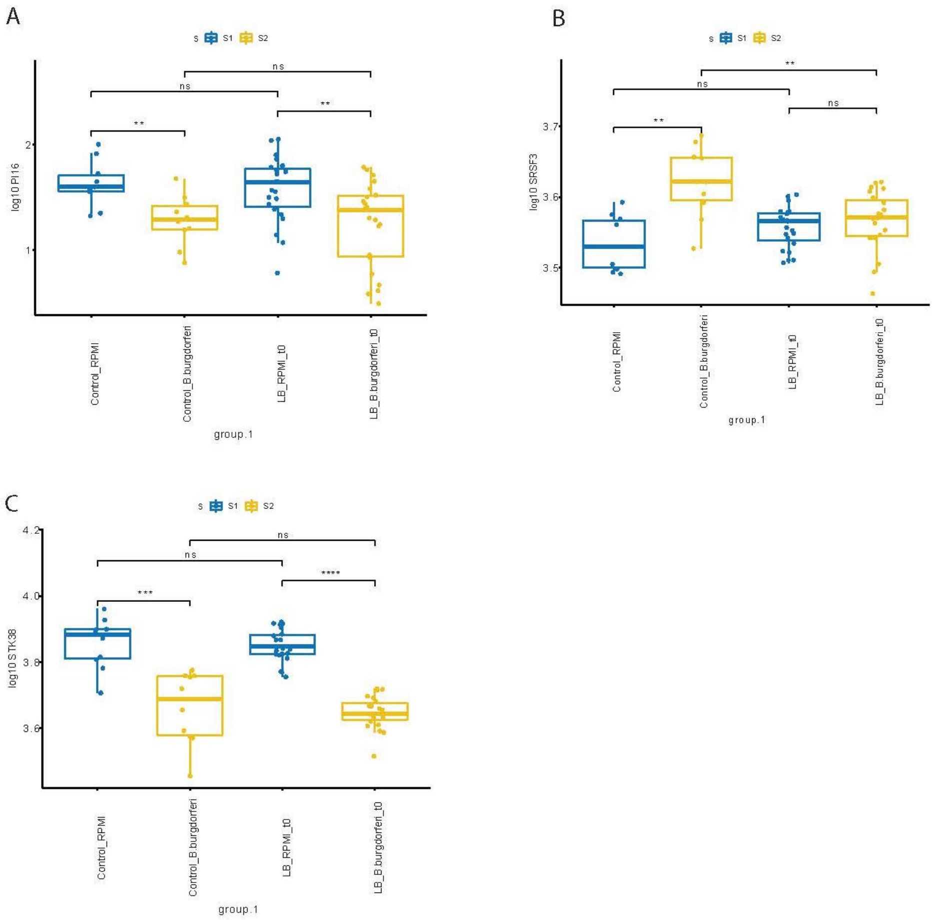

Supplementary figure 2. Expression of additional genes affected by rs1061632.

(A) Lower expression of *PI16* in PBMCs stimulated with *B. burgdorferi* s.l. compared to unstimulated PBMCs, both in LB patients and in healthy controls.

(B) Higher expression of *SRSF3* was only observed in *B. burgdorferi* s.l. stimulated PBMCs from healthy controls.

(C) Expression of *STK38* was lower in *B. burgdorferi* s.l. stimulation than in unstimulated PBMCs, both in LB patients and in healthy controls.

Pairwise comparison p-values represent the result of Wilcoxon rank sum test. * p<0.05, ** p<0.01, *** p<0.001, **** p<0.0001, ns = non-significant.
